# IMNM: integrated multi-network model for identifying pepper leaf diseases

**DOI:** 10.3389/fpls.2025.1558349

**Published:** 2025-09-12

**Authors:** Zhaopeng Cai, Nadia Farhana, Asif Mahbub Karim, Fengyan Zhai, Wenwen Huang, Meng Guo

**Affiliations:** ^1^ School of Computer and Data Science, Research Center of Smart City and Big Data Engineering of Henan Province, Henan University of Urban Construction, Pingdingshan, China; ^2^ Binary Graduate School, Binary University of Management & Entrepreneurship, Puchong, Selangor, Malaysia; ^3^ Department of Business Administration, Stamford University Bangladesh, Dhaka, Bangladesh; ^4^ Department of Plant Pathology, Henan Province Engineering Research Center of Biological Pesticide & Fertilizer Development and Synergistic Application, Henan Institute of Science and Technology, Xinxiang, China; ^5^ College of Agricultural Resources and Environment, Henan Institute of Science and Technology, Xinxiang, China

**Keywords:** integrated, deep learning, multi-network model, identifying, pepper leaf diseases

## Abstract

As a vegetable crop with high economic value, the yield of pepper is often significantly restricted by leaf diseases, and the spots formed by these diseases on the surface of leaves are highly complex in color and texture characteristics. To overcome the shortcomings of traditional manual identification methods, such as low efficiency, time-consuming, and labor-consuming, an integrated multi-network model (IMNM) was established by combining an improved ResNet, a dynamic convolution network (DCN), and a progressive prototype network (PPN), which was aimed at five typical pepper leaf samples (healthy, virus, leaf blight, brown spot, and phyllosticta). The experimental results show that IMNM achieves 98.55% accuracy in pepper disease identification, which is significantly better than the benchmark models such as Inception-V4, ShuffleNet-V3, and EfficientNet-B7. In the cross-species generalization verification, the average identification accuracy of the model for apple, wheat, and rice leaf diseases increased to 99.81%, and its four core indicators of specificity, precision, sensitivity, and accuracy were all stable over 98%. This demonstrates that IMNM can effectively analyze the color and texture characteristics of highly heterogeneous disease spots and possesses strong cross-crop generalization capabilities. Its technical path lays a theoretical foundation for the development of field mobile disease diagnosis equipment based on deep learning, and is of great value for promoting the engineering application of an intelligent monitoring system for crop diseases and insect pests.

## 1 Introduction

Pepper is an important vegetable crop. Leaf diseases and insect pests affect the growth of the pepper (Shaheen et al., 2021). These diseases cannot be eradicated, but they can be treated and monitored to reduce their impact.

A multitude of biotic and abiotic stressors limit crop productivity. Visible pests and microorganisms are the two types of biotic stress conditions (Bhar et al., 2022). The most apparent pests are insects, but rodents and animals can also cause agricultural harm. Abiotic stress (Omae and Tsuda, 2020) refers to physical issues such as poor environmental conditions and chemical stress in soil, air, and irrigation water caused by toxic circumstances. According to the Food and Agriculture Organization of the United Nations, pests and diseases account for 20 to 40% of global food production losses. As a result, diseases can cause major economic and environmental losses in agricultural product quantity and yield (Savary et al., 2019).

Currently, precision agriculture is utilized to combine multiple information technologies to optimize agricultural yields while minimizing expenses and agricultural losses (Shaikh et al., 2022). Artificial intelligence, image processing, and sensor networks are some examples of these information technologies. As a result, one of the most commonly used technologies for crop disease identification is image processing. Its primary objective is to locate signs of plant pathogens. The distinct visual damage modes that each disease causes in plant tissue may be due to issues with image processing and artificial intelligence systems. The same algorithm might not always be able to distinguish between many diseases.

This study proposed a novel deep learning method, the IMNM, for identifying pepper leaf disease to fix the aforementioned flaws. The following are the primary contributions of this paper:

1. To address gradient vanishing and network deterioration in the suggested model, this study presents an improved ResNet solution and uses a quick connection channel between input and output features to prevent information omission.
2. To improve the ability to express features of the model, this study employs the DCN and adaptively chooses the best convolutional kernels for feature extraction.
3. To enhance the training effectiveness of the model and shorten the training time of the model, this study adopts the PPN.

The remainder of the paper is structured as follows: An overview of the literature on crop leaf disease identification is presented in Section 2. A summary of the mathematical theory of the proposed framework is given in Section 3. The experimental evaluation metrics and discussion to support the suggested system are the main topics of Section 4. Section 5 concludes the paper by outlining some suggestions for further investigation.

## 2 Literature reviews

Traditional machine learning techniques and well-liked deep learning algorithms are two different sorts of strategies for identifying crop diseases with the development of artificial intelligence.

Traditional machine-learning image processing methods, as reported by Wang et al. (2022), typically utilize color, shape, texture, and other information to generate feature vectors, which are then classified using support vector machines. Chaudhari and Patil (2023) applied genetic algorithms to image segmentation and conducted research using ensemble models, with identification accuracy exceeding 92% compared to individual SVM, Naive Bayes, and KNN classifiers. Islam et al. (2024) used image processing techniques and support vector machines to classify stress symptoms of early pepper seedlings with an accuracy of 85%. Rusliyawati et al. (2024) proposed a model for pepper leaf disease identification based on a radial basis function neural network, and the research showed that the identification accuracy of this model was 91.67%. Fakhrurroja et al. (2024) developed a model for pepper leaf disease classification based on support vector machine technology, and the identification accuracy of the model was 92.13%. Valderrama Solis et al. (2025) proposed several identification models based on XGBoost, SVM, and decision tree algorithms, and researched Aleurothrixus foccosus in citrus. The results showed that the accuracy rates of the XGBoost, SVM, and decision tree models were 82%, 75%, and 65%, respectively. Kim et al. (2025) used support vector machines, logistic regression, and neural networks to rapidly and accurately identify novel variants of tomato spotted wilt virus, with identification accuracy rates of 71%, 79%, and 84%, respectively. Deep learning algorithms outperform conventional machine learning techniques in detecting agricultural diseases and pests, as demonstrated by prior research. Gautam et al. (2022) developed a hybrid CNN model for classifying paddy leaf diseases, achieving 96.4% accuracy. Palve (2023) combined deep learning, computer vision, and Tensorflow techniques to achieve 90.1% accuracy in leaf disease identification experiments such as sweet pepper, tomato, and potato. Dai et al. (2023) proposed an enhanced lightweight model based on the GoogLeNet architecture to identify pepper leaf diseases with an accuracy of 97.87%. To solve the problems of low accuracy and insufficient image representation ability in previous research on spinach leaf disease identification, Xu et al. (2024) proposed a new model combining a spatial attention mechanism and nucleated bilinear aggregation technology. The identification accuracy of this model reached 95.12. Wang Y. et al. (2024) conducted experiments on potato, corn and tomato leaf datasets with mask autoencoder and convolutional block attention module, and their average identification accuracy reached 95.35%. Wan et al. (2024) proposed a novel pepper disease identification model based on TPSAO-AMWNet, with an average identification accuracy of 93.52%. Rusliyawati et al. (2024) developed a pepper leaf disease identification model based on image analysis using radial basis function neural networks, and the identification accuracy of the model reached 91.67%. Sultan et al. (2025) proposed an improved Xception architecture to identify leaf diseases of roses, mangoes, and tomatoes, which achieved a leaf disease identification accuracy rate of 98.00%. Ashurov et al. (2025) combined Depthwise CNN with SE block and residual jump join to propose an automatic plant disease identification method, achieving 96% identification accuracy.

To sum up, there are obvious technical bottlenecks in current crop disease identification research: traditional machine learning relies on artificial features and shallow classifiers, although it achieves moderate accuracy in limited scenarios, its feature expression ability is limited and its generalization is insufficient; although deep learning methods significantly improve accuracy, they still face three challenges: insufficient feature representation of single network, high parameter quantity restricting deployment efficiency and weak cross-crop adaptability. Therefore, this study proposes a lightweight framework that integrates improved ResNet (optimized gradient propagation), DCN (adaptive kernel parameter selection), and PPN (momentum prototype iteration) to enhance the ability of complex lesion feature extraction through a multi-module cooperation mechanism, while reducing computational costs and improving cross-domain generalization performance. The follow-up experiments will systematically evaluate the validity of the model from theoretical verification, ablation analysis, cross-crop migration, and many other aspects based on five kinds of pepper leaf data, to provide a new paradigm for agricultural intelligent diagnosis with both accuracy and practicality.

## 3 Proposed process of the IMNM

### 3.1 Improved ResNet

#### 3.1.1 Definition of the ResNet

The ResNet is a deep Residual Network first proposed by Kaiming He’s team in 2015 in He et al. (2016), which was included as an oral presentation in CVPR2016, the premier conference on computer vision in the world. He et al. (2016) pointed out that the ResNet is a deep convolutional neural network architecture whose core mechanism is to pass shallow features directly to deeper layers through cross-layer residual connections, forcing the network to learn residual mappings of inputs and outputs rather than original feature mappings.

#### 3.1.2 Fundamentals of the ResNet

The depth of deep neural networks is the most direct way to improve network performance. As the number of layers increases, the vanishing gradient will become more and more obvious in the process of regression, and the corresponding network training effect will become worse.

These issues were addressed by the outstanding ResNet (Bhuma and Kongara, 2022), which added a shortcut connection between an input and output channel. In this way, the input information can directly reach the output, avoiding the omission of information. When the network was trained, it only needed the output residual.

The residual function can significantly increase the number of network layers and is simpler to tune. The residual unit looks like Equation 1:


(1) $$F(x)=W_{2}\sigma (W_{1}x)$$

where σ is ReLU. $W_{1}$ and $W_{2}$ represent the weights. Then it obtains an output *H* (*x*) through a shortcut and the second ReLU. *H* (*x*) can be computed as Equation 2:


(2) $$H(x)=F(x,\left\{W_{i}\right\})+x$$

When *H* (*x*) changes the dimensions of the input and output, it can perform a linear transformation on *x* during the shortcut operation. It is Equation 3:


(3) $$H(x)=F(x,\left\{W_{i}\right\})+W_{s}x$$

#### 3.1.3 Details description and implementation code of the improved ResNet

##### 3.1.3.1 Details description of the improved ResNet

**Figure 1:**
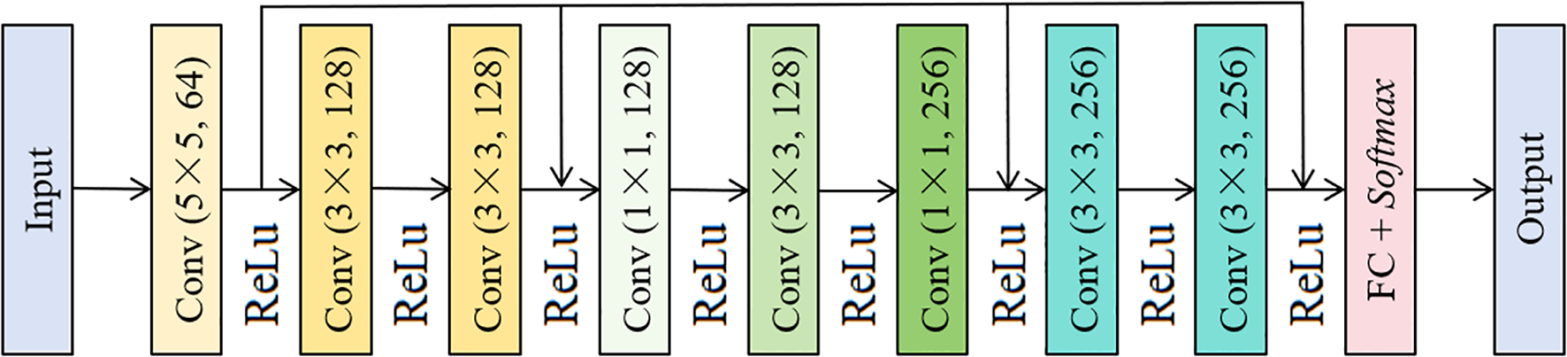
Improved residual unit architecture.

A single residual unit cannot improve the performance of networks, so this study designed three residual units. The specific structure is described in **Figure 1**.

As shown in **Figure 1**, the details of the improved ResNet mainly include: The improved ResNet network takes 256×256 resolution RGB image as input, the first layer extracts 64 channel features (output size 128×128) through 5×5 convolution kernel (step 2, fill 2), and generates input feature map (InputResNetData) after batch normalization (BN) and ReLU activation. The feature map enters the first residual block processing: the main path contains two 3×3 convolution layers, the first layer convolution (output 128 channels, step 2, fill 1) reduces the feature map size to 64×64, the second layer convolution (fill 1) maintains the resolution of 64×64; the jump connection adjusts the input dimension through 1×1 convolution (output 128 channels, step 2), and the main path and the jump connection result are added to output 128 channels 64×64 feature map (OutResNet1) through ReLU activation. The main path of the second residual block is firstly fused with cross-channel information through 1×1 convolution (input and output are both 128 channels) to enhance the feature expression ability, then downsampled to 32×32 through 3×3 convolution (step size 2, filling 1), and then expanded to 256 channels through 1 × 1 convolution; the skip connection adjusts the dimension through 1×1 convolution (output 256 channels, step size 2), and outputs 256-channel 32×32 feature map (OutResNet2) after addition activation. The main path of the third residual block processes the input features through two 3×3 convolutional layers (padding 1), maintaining 256 channels and 32×32 resolution. Since the main path output (256 channel 32×32) and the skip connection input (OutResNet2, 256 channel 32×32) have the same number of channels, there is no need to adjust the dimension through a 1×1 convolution. The main path result and skip connection input are directly added and activated by ReLU, and the output 256-channel 32×32 feature map (OutResNet3) is output. The final features are compressed into 256-dimensional vectors by global average pooling (GAP), mapped to a 5-dimensional class space by fully connected layers, and probability distributions are generated by the Softmax function.

##### 3.1.3.2 Implementation code of the improved ResNet

For a link to the implementation code for the improved ResNet, please refer to the “Data availability statement” section of this paper.

#### 3.1.4 Analysis of contributions of introducing the improved ResNet

The introduction of the improved ResNet not only addresses the gradient disappearance problem mentioned above but also enhances the feature extraction capabilities of the network. Furthermore, the improved ResNet can extract multi-scale depth features, allowing it to capture both the overall structure and local textures of the blade. More specifically, in the task of leaf feature extraction and classification, the improved ResNet effectively extracts local texture and shape features of leaves, providing essential representations for subsequent classification.

### 3.2 DCN

#### 3.2.1 Definition of the DCN

The DCN is short for the dynamic convolution network, which was first presented by Yinpeng Chen’s team in 2019 in Chen et al. (2020), a paper received as an oral presentation by CVPR2020, the top international conference in the field of computer vision. Chen et al. (2020) pointed out that the DCN is an adaptive feature extraction framework, and its core mechanism is: in the convolutional layer, multiple parallel convolution kernels are dynamically aggregated through the input-dependent attention mechanism to form an optimal convolution operation for a specific input mode.

#### 3.2.2 Fundamentals of the DCN

**Figure 2:**
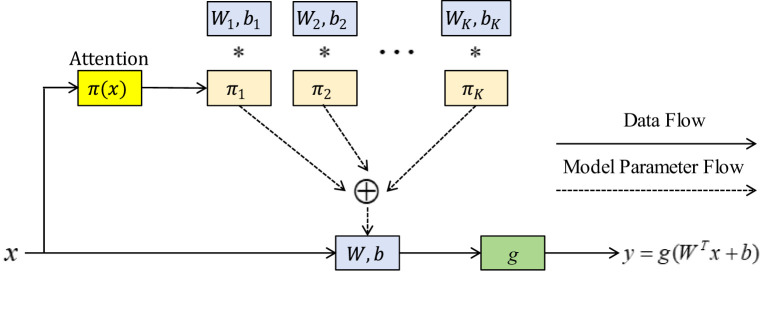
Dynamic perceptron architecture; The symbol * represents multiplication in mathematical operations.

With the help of dynamic convolution (Han et al., 2022), network architecture and computing requirements can be balanced. Without expanding the depth or width of the network, it improves the capacity for expression of the model. Instead of using a single convolution kernel for each layer, the DCN dynamically aggregates many parallel convolution kernels, modifies the weight of each convolution kernel, and chooses the right parameters in **Figure 2**.

In **Figure 2**, an output can be expressed Equations 4-7:


(4) $$y=g(W^{T}x+b)$$

(5) $$W=\sum_{k=1}^{K}\pi_{k}(x)W_{k}$$

(6) $$b=\sum_{k=1}^{K}\pi_{k}(x)b_{k}$$

(7) $$\sum_{k=1}^{K}\pi_{k}(x)=1,0\leq \pi_{k}(x)\leq 1$$

where *W, b, and g* are the weight, offset, and activation functions, respectively. $\pi_{k}$ represents an attention weight. It is not fixed but changes with an input. $\pi_{k}$ includes the attention weight calculation and dynamic weight fusion. Its process can be formulated according to Equation 8:


(8) $$O(W^{T}x+b)\gg O(\sum \pi_{k}b_{k})+O[\pi (x)]$$

where O(·) is the calculation amount of perceptron.

Three kernels with the same scale and channel were set after a dynamic convolution, and they were fused using their respective attention weights to produce the convolution kernel parameters. The GAP is used in the attention layer to get global spatial characteristics. Softmax normalizes two fully connected (FC) layers that are mapped to three dimensions so that the obtained attention weight can be distributed among the three kernels of this layer. The previously fixed convolution kernel can now be dynamically chosen depending on the input, which greatly enhances the ability of feature expression.

#### 3.2.3 Details description and implementation code of the DCN

##### 3.2.3.1 Details description of the DCN

**Figure 3:**
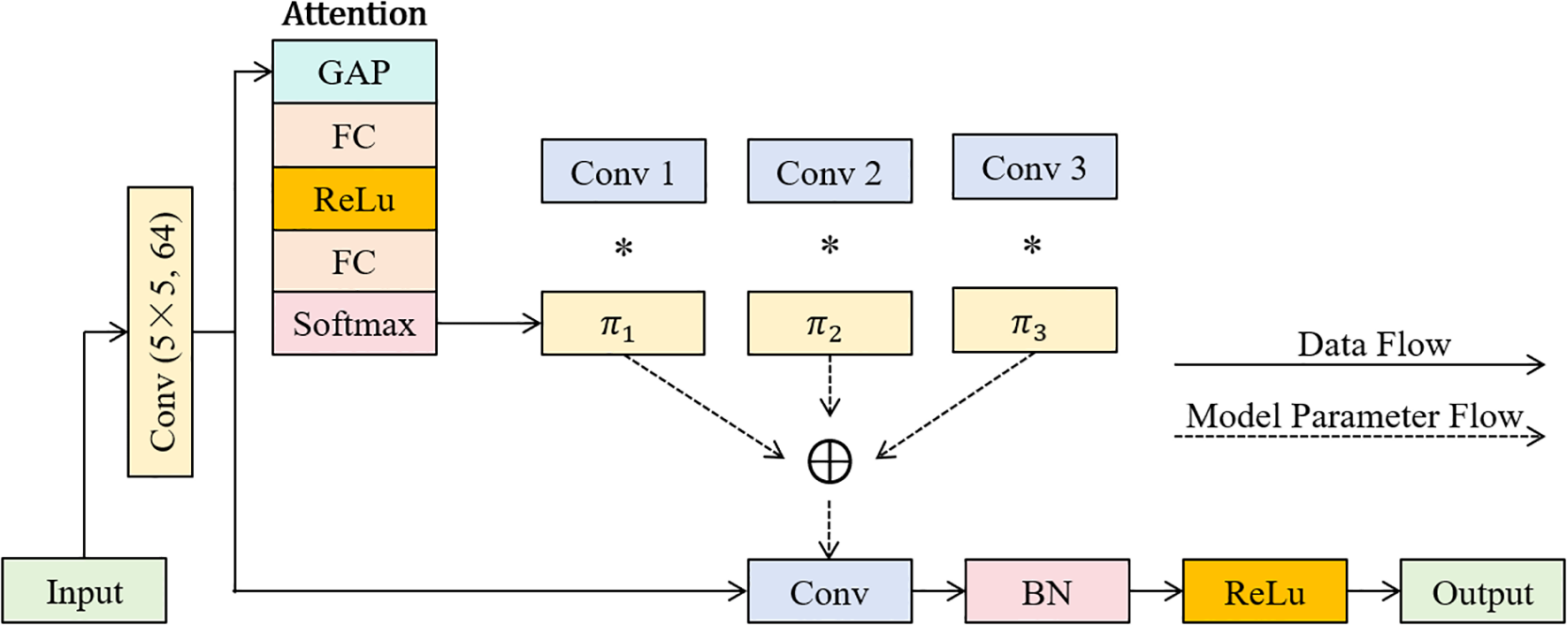
The DCN used in this study; The symbol * represents multiplication in mathematical operations.

The DCN used in this study has three convolutional kernels in **Figure 3**.

As shown in **Figure 3**, the details of DCN in this study mainly include: First, DCN takes 256 × 256 resolution RGB image as input, extracts 64 channel features (size 128 × 128) through a 5 × 5 convolution kernel (step 2, fill 2), and then obtains the input data to enter the attention mechanism (InputAttentionData) after batch normalization and ReLU activation. The flow process of InputAttentionData in the attention mechanism is as follows: the GAP is performed on InputAttentionData to extract global spatial features of input features, so that the model can capture key information from the overall level; Then, the output of the GAP is passed into an FC layer for calculation, activated by ReLu, and then passed into another FC layer for calculation. After that, the result is normalized by the Softmax function, thus obtaining dynamically changing attention weights (Attention_weights). These weights reflect the degree of association between input features and convolution kernels, and are not fixed and will adjust with input. Then, the network initializes three same-scale 3 × 3 convolution kernels (input 64 channels, output 256 channels, step 1, fill 1), and performs channel-level weighted fusion on convolution kernel parameters according to Attention_weights. Then, the fused dynamic convolution kernel is applied to InputAttentionData (step 2, fill 1) to output a 256-channel 64 × 64 feature map, which is downsampled to 32 × 32 by 3 × 3 maximum pooling (step 2), and then reduced to 16 × 16 resolution by 2 × 2 average pooling. The final features are batch normalized and ReLU activated to output a 256-channel 16 × 16 enhanced feature map.

##### 3.2.3.2 Implementation code of the DCN

For a link to the implementation code for the DCN, please refer to the “Data availability statement” section of this paper.

#### 3.2.4 Analysis of contributions of introducing the DCN

After introducing the DCN, it can enhance the adaptability to the deformation of disease spots and extract dynamic local features. By dynamically generating convolution kernel parameters, the model gains the ability to adaptively adjust the receptive field according to the input characteristics. This approach significantly improves the capacity to capture multi-scale disease features of the model and overcomes the limitations of traditional fixed convolution kernels regarding scale sensitivity. Its dynamic characteristics are particularly beneficial for addressing the morphological diversity of disease spots caused by the change of shooting angles and illumination in leaf images.

### 3.3 PPN

#### 3.3.1 Definition of the PPN

The PPN is short for the progressive prototype network, which was first proposed by Chaoqun Wang’s team in Wang et al. (2021) in 2021, which was officially included as a long article by NeurIPS2021, the top international conference in the field of machine learning. Wang et al. (2021) pointed out that the PPN is a dynamic feature alignment framework, and its core mechanism is to gradually enhance the cross-domain transferability and category discrimination of visual features by alternately optimizing attribute prototypes and category prototypes.

#### 3.3.2 Fundamentals of the PPN

This study creates the PPN, which primarily consists of a convolution layer, a BN layer, a max-pooling layer, an FC layer, and a softmax layer, to increase training efficiency and shorten training time. This study determines the prototype feature representation for each category following feature extraction. The average value of all sample features in the validation set of the category makes up each category prototype. The precise calculation formula is Equation 9.


(9) $$c_{k}=\frac{1}{\left|S_{k}\right|}\sum_{(x_{i},y_{i})\in S_{k}}f(x_{i})$$

where $c_{k}$ is the prototype feature set for each category $\left|S_{k}\right|$ represents the number of all samples belonging to category *k* in the validation set, and $f(x_{i})$ is the feature vector obtained from feature extraction of training samples $x_{i}$ .

By comparing the Euclidean distance and normalized exponential function between each sample and the category prototype in the query set, the Euclidean distance is converted into probability, and the probability distribution of the test sample $\hat{x}$ for category *k* is obtained. The probability can be defined as Equation 10:


(10) $$p_{\theta }(\hat{y}=k|\hat{x})=\frac{\exp(-d(f(\hat{x}),c_{k}))}{\sum_{k^{\prime }}\exp(-d(f(\hat{x}),c_{k^{\prime }}))}$$

where d(x) is used to solve the Euclidean distance, θ represents parameters, and $k^{\prime }$ is the order number of a category.

The PPN uses the cross-entropy function as the loss function during training, and its expression is as Equation 11:


(11) $$L(\theta )=-\sum_{\hat{x}}y\log(p_{\theta }(\hat{y}|\hat{x}))$$

where *y* is the true label of a sample and $\hat{y}$ represents a prediction label.

#### 3.3.3 Details description and implementation code of the PPN

##### 3.3.3.1 Details description of the PPN

Now assuming that the PPN in this study takes a feature map with dimension [B,256,32,32] as input, the details of DCN mainly include: first, PPN uses a 2×2 convolution kernel (256 channels, step 1, fill 1) to enhance the input features locally, and the output dimension remains [B,256,32,32]; then the feature distribution is normalized by batch normalization layer, and nonlinear response is introduced via ReLU activation function. The maximum pooling layer (2×2 kernel, step size 2) compresses the spatial resolution of the feature map from 32×32 to 16×16 (dimensions [B,256,16,16]) to focus on significant areas and reduce computational complexity. The GAP further aggregates spatial features into a 256-dimensional global vector ([B,256]), capturing the overall semantic information of the image. The fully connected layer linearly maps the vector to a 5-dimensional space, directly corresponding to five classes of objects: Brown spot, Leaf bright, Healthy, Phyllosticta, and Virus. In the training phase, the network dynamically maintains five 256-dimensional prototype vectors (the initial value is zero vector), extracts the feature mean of the current same sample from each batch as a temporary prototype, and iteratively optimizes the prototype parameters through a momentum update mechanism, wherein the stability of the update process is guaranteed by setting momentum coefficients. In the inference stage, the Euclidean distance matrix between input eigenvectors and prototypes is calculated, and the Softmax function normalizes the 5-dimensional probability distribution according to the class similarity score.

##### 3.3.3.2 Implementation code of the PPN

For a link to the implementation code for the PPN, please refer to the “Data availability statement” section of this paper.

#### 3.3.4 Analysis of contributions of introducing the PPN

After introducing the PPN, it strengthens classification decisions through prototype comparison and improves the generalization capabilities of the model. The specific workflow of the PPN involves a two-stage prototype learning strategy: the first stage constructs a discriminative feature space between classes, while the second stage dynamically optimizes the classification boundaries. The progressive optimization mechanism of the PPN helps mitigate the class imbalance problem and prevents the model from overfitting to high-frequency disease classes.

### 3.4 Details description and implementation code of the IMNM

#### 3.4.1 Details description of the IMNM

**Figure 4:**
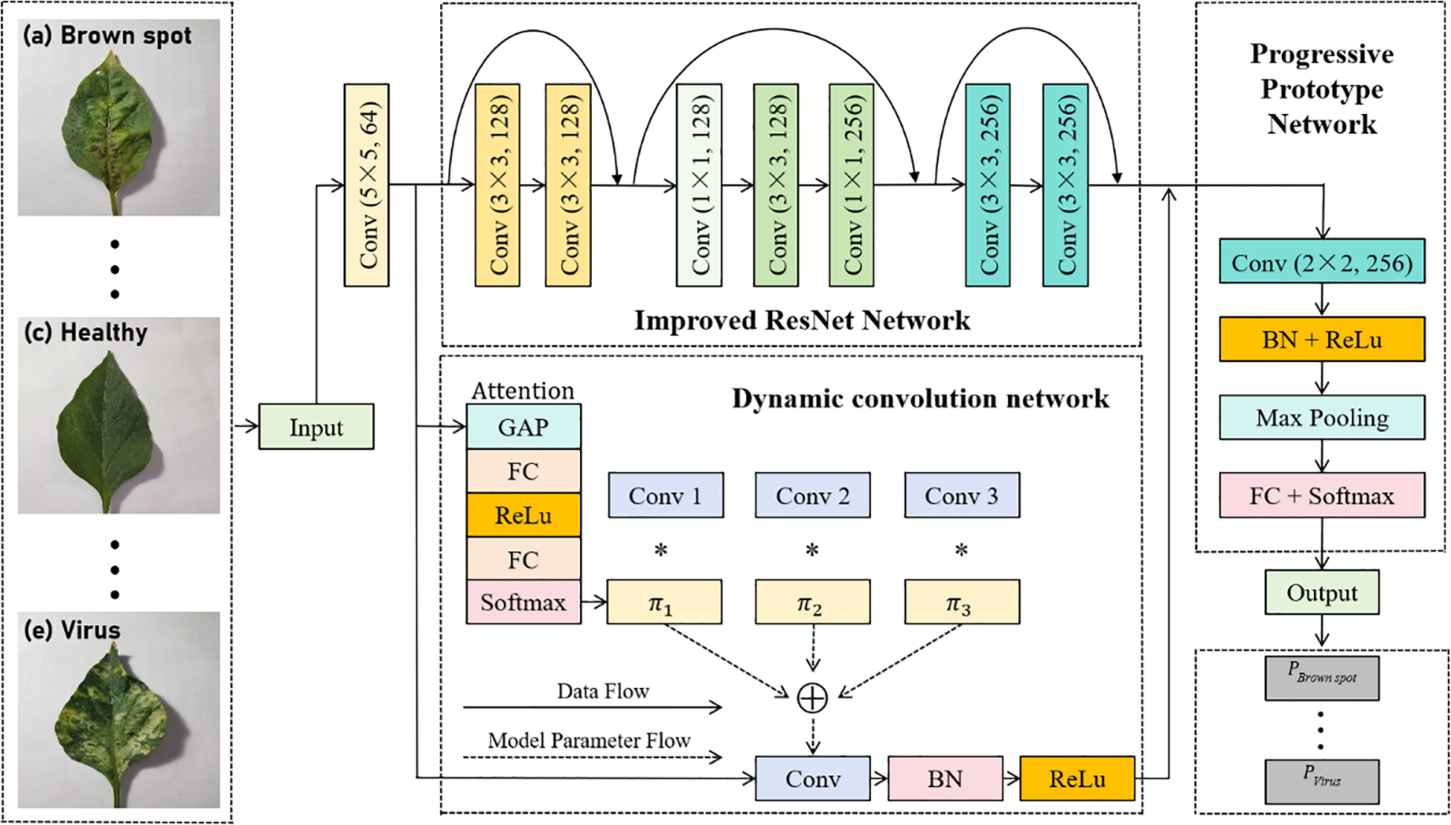
Overall identification framework of the IMNM; ‘(a) Brown spot’ represents the disease name of the leaf in the first picture; ‘(c) Healthy’ means that the leaf in the third picture is disease-free; ‘(e) Virus’ represents the disease name of the leaf in the fifth picture; The symbol * represents multiplication in mathematical.

The IMNM consists mainly of the improved ResNet, the DCN, and the PPN. **Figure 4** depicts the overall identification framework intuitively.

As shown in **Figure 4**, the IMNM sequentially performs feature extraction, multiscale fusion, and classification. The improved ResNet processes 256×256 RGB inputs through three residual blocks: a 5×5 initial convolution (64 channels, stride 2) followed by cascaded blocks (128→256 channels) with skip connections, outputting 256-channel 32×32 features. The DCN applies a 5×5 convolution (64 channels) and attention-guided fusion of three 3×3 kernels to generate 256-channel 16×16 features via dynamic convolution and pooling. The PPN enhances these features via 2×2 convolution, max-pooling to 16×16, and pooling to 256D vectors. Prototype vectors are momentum-optimized during training, generating 5D probabilities via Softmax. The model achieves end-to-end processing from 256×256 inputs to disease-specific probability distributions.

#### 3.4.2 Implementation code of the IMNM training

For a link to the implementation code of the IMNM training, please refer to the “Data availability statement” section of this paper.

### 3.5 Analysis of the combination advantage of the IMNM subnets

The IMNM realizes the integration of multi-scale features, enhancement of dynamic adaptability, and optimization of computational efficiency. The specific analysis is as follows:

#### 3.5.1 Integration of multi-scale features

First, the improved ResNet extracts global semantic features of lesions through a deep network and establishes high-level abstract representations. Second, the DCN embeds dynamic convolution in the shallow and middle feature layers of the ResNet, adaptively adjusts the receptive field according to the size and texture complexity of local lesions, and accurately captures the details and morphological variations of small lesions. Finally, the PPN performs prototype projection on multi-scale features at the end, fusing local details (the DCN output) and global semantics (the ResNet output) into hierarchical feature pyramids by contrast loss constraint feature space geometry. The three cooperate to form the integration of multi-scale features of the IMNM and finally realize efficient mapping from pixel-level texture to disease category.

#### 3.5.2 Dynamic adaptive enhancement

First, the improved ResNet filters out key lesion features through residual structure, providing high discriminative input for subsequent processing. Second, the DCN dynamically generates convolution kernels based on geometric characteristics of lesions to accurately capture local deformation features. Finally, the PPN constrains feature spatial distribution through prototype comparison learning to ensure the statistical separability of different diseases. The closed-loop optimization mechanism makes the IMNM not only maintain local sensitivity but also maintain cross-domain classification stability through global constraints, thus enhancing the dynamic adaptability of the IMNM.

#### 3.5.3 Increase in computational efficiency

First, the improved ResNet multiplexes cross-layer features through residual hopping connections to reduce redundant computation in deep networks. Second, the DCN dynamically generates sparse convolution kernels based on local characteristics of input lesions, activating complex operations only in lesion regions to avoid global computational resource waste. Finally, the PPN compresses feature space dimensions by using prototype contrast learning, significantly reducing computational complexity in classification layers. The improved ResNet, DCN, and PPN coordination mechanism effectively improves computational efficiency while ensuring accuracy.

## 4 Experimental results and analysis

### 4.1 Experimental environment

The network model proposed in this paper employs the PyTorch 2.3.1 deep learning framework with CUDA 11 and cuDNN 8.9 acceleration libraries for GPU-accelerated training. It is implemented in Python 3.9 using PyCharm 2024.2.3. The computational infrastructure includes a 14-core Intel Ultra 5 CPU@3.00 GHz and an NVIDIA GeForce RTX 4060 GPU for parallel processing.

### 4.2 Details description of the dataset

#### 4.2.1 Collection object

The collected object images are collected from pepper fields in different planting areas of pepper planting base in Henan Province, China, covering leaves in different growth cycles, from seedling stage to mature stage, and the collection time span reaches one growth season, which ensures the diversity of samples in growth stage, environmental factors and other aspects, making the dataset more representative.

#### 4.2.2 Collection equipment and environment

A mobile phone camera (Honor Play 4, China) was used to shoot in the standardized laboratory. This camera has high resolution and excellent color reproduction ability, which can accurately capture leaf details.

#### 4.2.3 Collection techniques

Collect images of five types of pepper leaves (healthy, virus, leaf blight, brown spot, and leaf spot disease) taken from multiple angles under different lighting conditions, such as strong light, normal light and low light to simulate light changes in actual application scenarios and increase the authenticity and complexity of data.

#### 4.2.4 Image enhancement means

Due to the actual frequency of various diseases in the field, the sample size of some disease categories is still relatively small, so this study has to adopt image enhancement means such as generative adversarial networks in addition to actual image acquisition, to obtain a more balanced distribution of data resources.

#### 4.2.5 Image preprocessing

The original image is uniformly cropped to 256×256 resolution, which ensures that the details of leaf disease characteristics are preserved and reduces the consumption of computational resources.

#### 4.2.6 Data annotation

The professional guidance of plant pathology experts from authoritative scientific research institutions in the field of plant protection in Henan Province shall be provided throughout the data labeling work to ensure the high consistency, professionalism, and reliability of data labeling.

#### 4.2.7 Data samples

**Figure 5:**
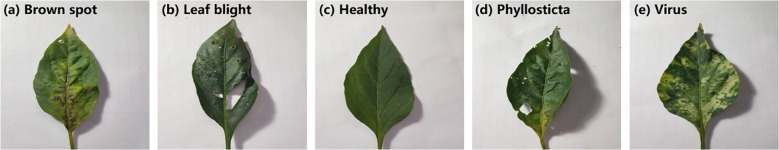
Five samples; ‘**(a)** Brown spot’ represents the disease name of the leaf in the first picture; ‘**(b)** Leaf blight’ represents the disease name of the leaf in the second picture; ‘**(c)** Healthy’ means that the leaf in the third picture is disease-free; ‘**(d)** Phyllosticta’ represents the disease name of the leaf in the fourth picture; ‘**(e)** Virus’ represents the disease name of the leaf in the fifth picture.

Data samples include one leaf sample for each of the five categories of Brown spot, Leaf bright, Healthy, Phyllosticta, and Virus, as shown in **Figure 5**.

#### 4.2.8 Datasets size and partitioning strategy

Through original acquisition and image enhancement, this study finally obtained a total of 11447 images. This study randomly divided the 11,447 samples into a training set, a validation set, and a test set in a 3:1:1 ratio. **Table 1** displays the results.

**Table 1:** The quantity of each category.

Types	Training set	Validation set	Test set	Total number
Virus	1502	503	503	2508
Healthy	1239	416	416	2071
Leaf blight	1322	445	445	2212
Brown spot	1437	483	483	2403
Phyllosticta	1347	453	453	2253
Total number	6847	2300	2300	11447

(9) Open access links to datasets

Open access links to datasets, please refer to the “Data availability statement” section of this paper, are available for free download and use by researchers.

### 4.3 IMNM performance analysis based on the accuracy and loss curve

#### 4.3.1 Hyperparameter configuration information (including training time)

To verify the performance of the IMNM, the hyperparameter configuration information, including training time, utilized in this study, is shown in **Table 2**.

**Table 2:** Hyperparameter configuration information (including training time).

Methods	Batch size	Learning rate	Epoch	Test loss	Test accuracy	Time cost (hours)
IMNN	16	0.001	50	0.3921	0.9087	8.2
	32	0.002	150	0.3156	0.9324	19.8
	64	0.002	250	0.2532	0.9521	31.5
	32	0.001	350	0.1879	0.9724	41.3
	32	0.001	425	0.1524	0.9855	49.8
	64	0.002	525	0.1637	0.9819	75.8
The experimental setting	32	0.001	425	—	—	—

According to the data in **Table 2**, the reasons for selecting the setting “Batch size=32, Learning rate=0.001, Epoch=425” as the experimental setting are as follows:

##### 4.3.1.1 Test loss

The test loss at the setting “Epoch=425 (0.1524)” is lower than the test loss at the setting “Epoch=350 (0.1879)”, although slightly higher than the test loss at the setting “Epoch=525 (0.1637)”, but the difference is not significant. This indicates that the model has converged well at 425 epochs, and further increasing the training period may contribute only marginally to the loss reduction.

##### 4.3.1.2 Test accuracy

At the setting “Epoch=425”, the test accuracy is 0.9855, which is the highest of the three. This shows that in this training cycle, the model has the strongest generalization capabilities and is better equipped to predict unseen data.

##### 4.3.1.3 Time cost

In terms of time cost, the setting “Epoch=425” takes 49.8 hours for training, which is longer than the setting “Epoch=350 (41.3 hours), but considering its higher accuracy and lower test loss, the extra time investment is worth it. At the same time, compared with the setting “Epoch=525”, it saves approximately 26 hours of training time, and the performance improvement is not significant.

Considering the test loss, test accuracy, and time cost, the settings “Batch size=32, Learning rate=0.001, Epoch=425” not only ensure the performance of the model but also consider the training efficiency. This setting achieved the highest test accuracy and was reasonable in terms of time cost, so it was selected as an experimental setting.

#### 4.3.2 The accuracy and loss curve

**Figure 6:**
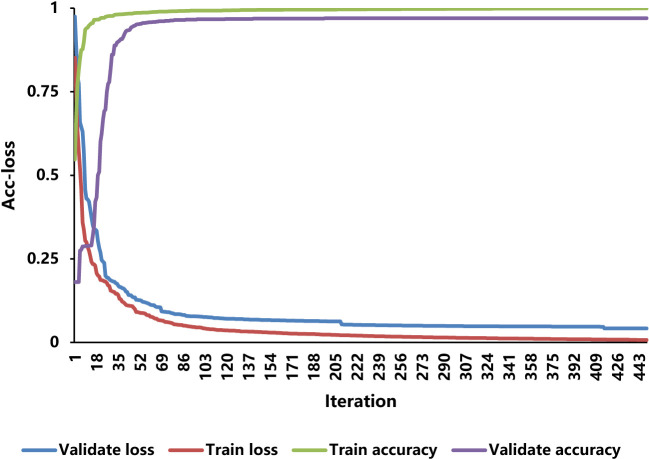
The Acc-loss curves for training and validation.

Through the training set and the validation set, this study trained the IMNM. **Figure 6** displays the variations in the accuracy and loss (Acc-loss) curve values.

The results of **Figure 6** show that as the iteration increases, the training loss and the validation loss decrease, while the training accuracy and the validation accuracy keep rising until saturation. As a result of these behaviors, the vanishing gradient and over-fitting do not occur during the training and validation processes.

### 4.4 Verification and analysis of the interpretability of the IMNM effectiveness

#### 4.4.1 Verification and analysis of interpretability based on convolution thermograms

**Figure 7:**
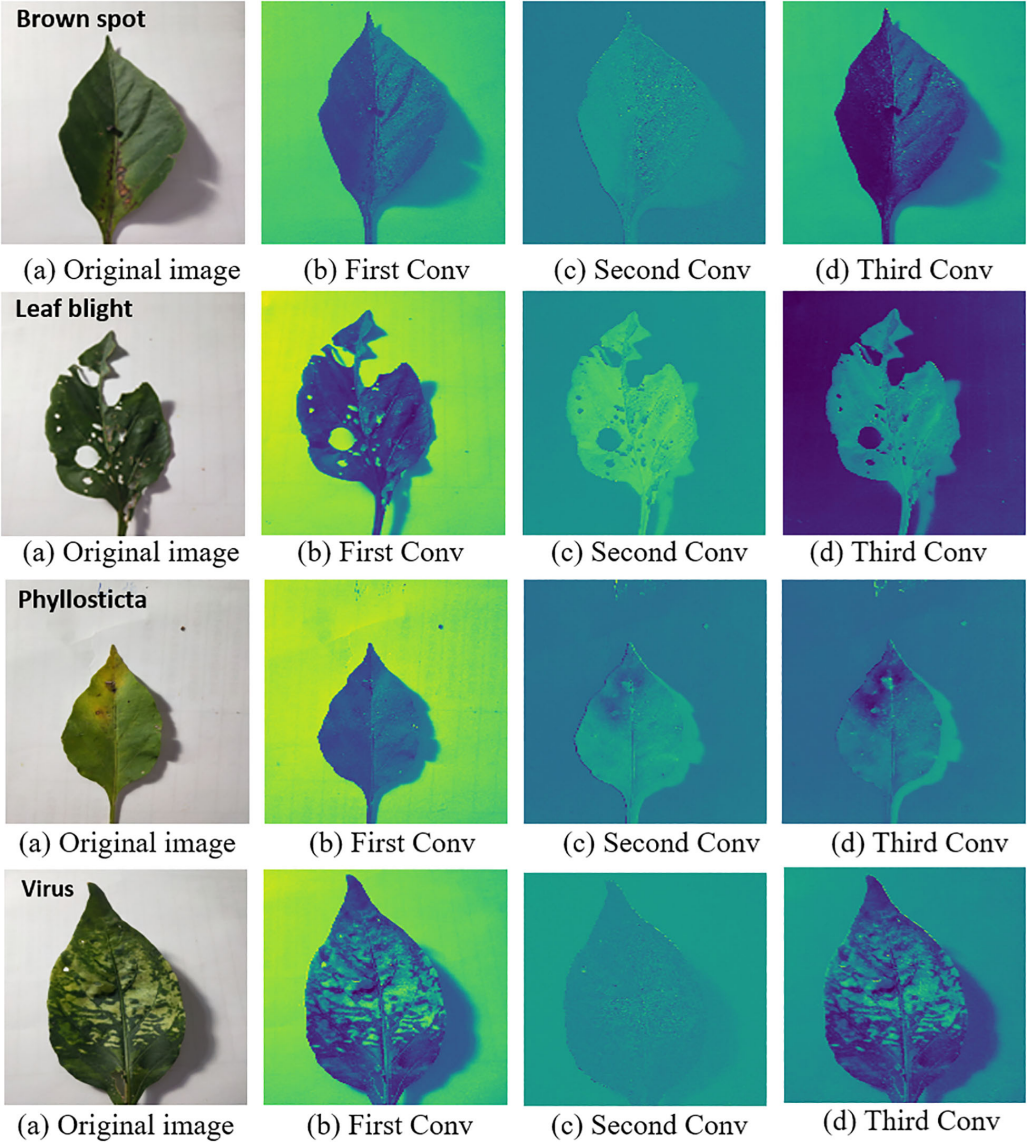
Heat maps of different layers with the IMNM; ‘Brown spot’ on the first picture in the first row represents the disease name of this leaf; ‘Leaf blight’ on the first picture in the second row represents the disease name of this leaf; ‘Healthy’ on the first picture in the third row means that the leaf is disease-free; ‘Phyllosticta’ on the first picture in the fourth row represents the disease name of this leaf; ‘Virus’ on the first picture in the fifth row represents the disease name of this leaf.

The IMNM correctly identifies key regions in the original image, in particular the diseased area on the diseased leaves, after comparing it with the heat map. This study displayed the heat maps of the test samples to verify the capacity to extract features of the IMNM. **Figure 7** displays the visual outcomes of the running IMNM.

The visual analysis shown in **Figure 7** verifies the ability of the IMNM model to accurately extract disease features. In the thermal map, the significant areas extracted by the IMNM completely covered the key features such as lesion edge, irregular shape, and vein abnormality. These areas clearly distinguished the lesion area from the background. Multi-stage convolutional activation patterns for different disease categories visually represent the feature learning process. For example, early convolutional layers focus on texture details, and deep networks integrate global semantics. This hierarchical feature activation mechanism not only verifies the effective capture of key disease features by the model but also reveals the biological basis of classification decisions through visual means, providing empirical support for the effective interpretability of the model.

#### 4.4.2 Analysis of interpretability based on inherent feature extraction ability of the IMNM subnetworks

##### 4.4.2.1 Improved ResNet

Feature maps of different layers of the improved ResNet, showing the extraction of the network from low-level textures to high-level semantics. In this study, the shallow network captures the vein texture, and the deep network identifies the shape and distribution pattern of the lesion, which reveals the hierarchical feature learning mechanism of the model. This just reflects the model to extract features of effective interpretability.

##### 4.4.2.2 DCN

The convolution kernel generated by the DCN adapts, and the convolution kernel shape changes triggered by different disease images. In the brown spot image, the dynamic convolution kernel may focus on the sharp spots, while in the leaf spot image, the convolution kernel may focus on the diffuse texture. This proves that the model can dynamically adjust the feature extraction strategy according to different disease features and enhance the technical reliability. This just reflects the model to extract features of effective interpretability.

##### 4.4.2.3 PPN

The PPN decodes the prototype vector of each disease category into representative image blocks, which show the similarity between the prototype and the input features. In this study, the prototype of brown spot disease may correspond to typical round brown spots, and the model completes classification by matching the input image with the prototype. The classification logic is explained by the “typical lesion template,” which is consistent with human cognitive habits. This proves the interpretability of the features extracted from the model.

#### 4.4.3 Analysis of the interpretability of generalization capabilities

The distribution of pepper, apple, wheat, and rice disease images in prototype space was compared to analyze how the model represented the disease spot characteristics of different crops uniformly. Brown spot disease may share similar prototypical matching patterns in pepper and rice leaves in the study. This can explain the underlying logic of the generalization ability of the model and enhance the credibility of multi-scenario applicability. This shows that the model has good generalization ability.

### 4.5 Formulas for the primary performance indicators of the IMNM

The specificity, precision, sensitivity, and accuracy were used to evaluate the IMNM. The evaluation metrics can be computed according to Equation 12-15.


(12) $$\text{Specificity}=\frac{TN}{TN+FP}$$

(13) $$\text{Precision}=\frac{TP}{TP+FP}$$

(14) $$\text{Sensitivity}=\frac{TP}{TP+FN}$$

(15) $$\text{Accuracy}=\frac{TP+TN}{TP+TN+FP+FN}$$

where TP and TN denote true positives (TP) and true negatives (TN). False positives (FP) and false negatives (FN) are abbreviated as FP and FN, respectively.

### 4.6 IMNM performance analysis based on the ablation experiments

#### 4.6.1 Result of the ablation experiments

**Figure 8:**
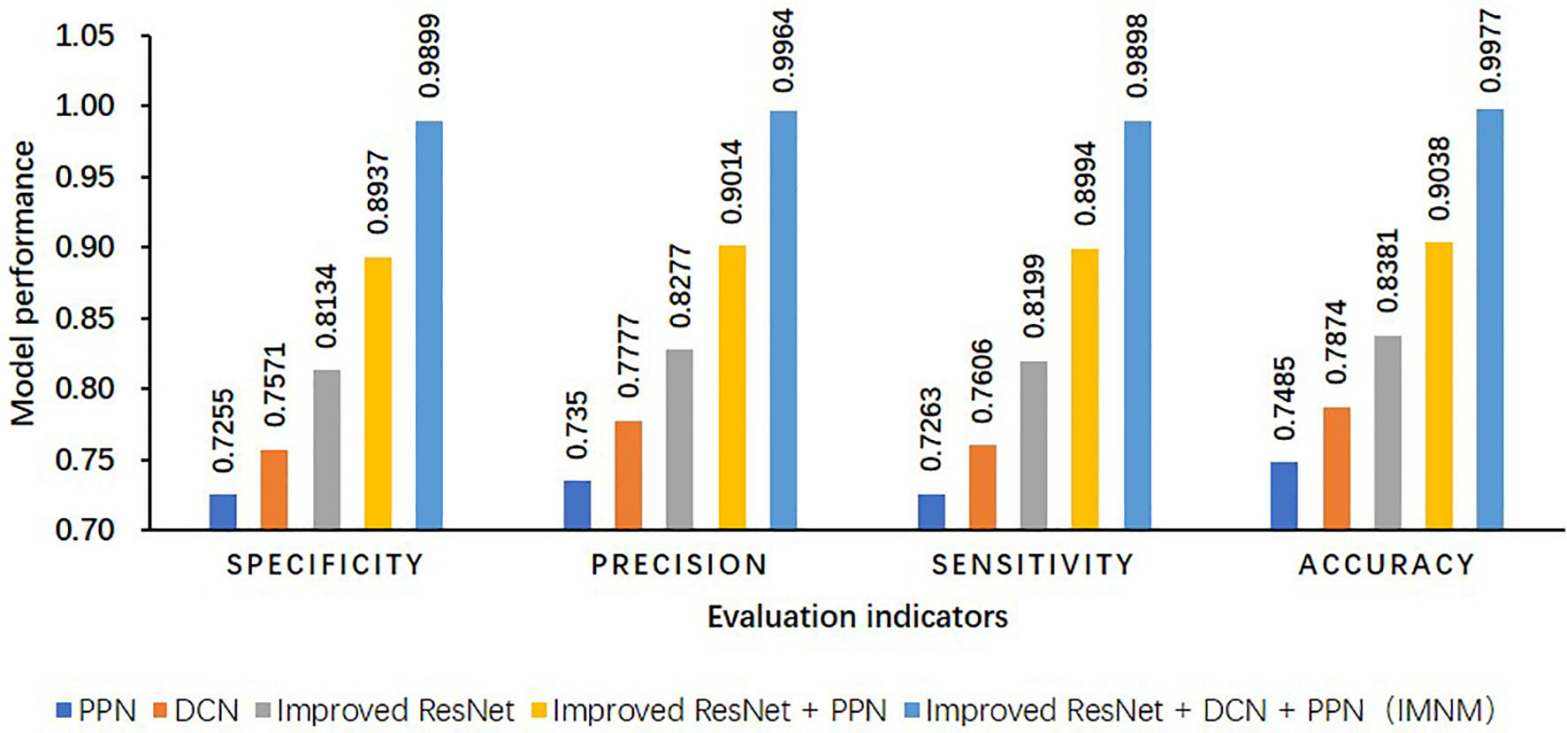
Results of ablation experiments on its own dataset of this study.

Because the IMNM made use of the improved ResNet, DCN, and PPN strategies, to validate the effectiveness of the improved strategies, this study conducted ablation experiments on its own dataset of this study in **Figure 8**.

The strategy based on model fusion provides several benefits over the single model and compensates for some of its drawbacks. In terms of accuracy, the IMNM outperforms the single PPN by 24.92%. In comparison to the single-network model structure, the IMNM is more stable and robust, and the IMNM successfully increases the categorization accuracy of samples of pepper sickness.

#### 4.6.2 Analysis of the result of the ablation experiments

##### 4.6.2.1 DCN

Using the DCN alone makes it easy to pay too much attention to local details due to a lack of deep semantic guidance, which will lead to limited classification performance due to a lack of semantic features, insufficient identification ability for complex diseases, and misjudgment of overall disease patterns by the DCN.

##### 4.6.2.2 PPN

Prototype learning is less sensitive to local texture details, and the PPN alone has insufficient ability to extract basic features, and the quality of feature extraction is easily disturbed by noise, which makes it difficult for the PPN to distinguish diseases with highly similar textures.

##### 4.6.2.3 Improved ResNet

As a basic model, its residual structure and multi-scale feature extraction ability provide a solid semantic feature basis for disease identification. However, due to the lack of adaptability of dynamic convolution to non-rigid deformation and the ability of prototype learning to capture fine-grained differences, its performance is limited in complex deformation lesions or fine classification, and classification decision boundaries are susceptible to background noise interference.

##### 4.6.2.4 Improved ResNet + PPN

In the deep network of the improved ResNet + PPN, the PPN prototype learning can maximize the separability of the leaf image feature space to enhance the classification discriminability of the model, so the enhanced model can exhibit stronger migration ability in cross-dataset testing, but the combination of the improved ResNet + PPN is weak in capturing dynamic deformation features, and the DCN can improve this defect.

##### 4.6.2.5 Improved ResNet + DCN + PPN

In the deep network of the improved ResNet + DCN + PPN, the DCN and PPN act on the ResNet backbone network from the feature generation dimension and feature constraint dimension, respectively, forming the closed-loop optimized link. The multi-scale perception of the DCN provides a richer basis for prototype construction for the PPN, while the discriminative constraints of the PPN, in turn, direct the DCN to focus on local regions with more classification value. Such multi-modal fusion and dynamic adaptability synergize to significantly improve the ability to identify complex diseases.

### 4.7 Comprehensive performance analysis of the IMNM

#### 4.7.1 Confusion matrix

**Figure 9:**
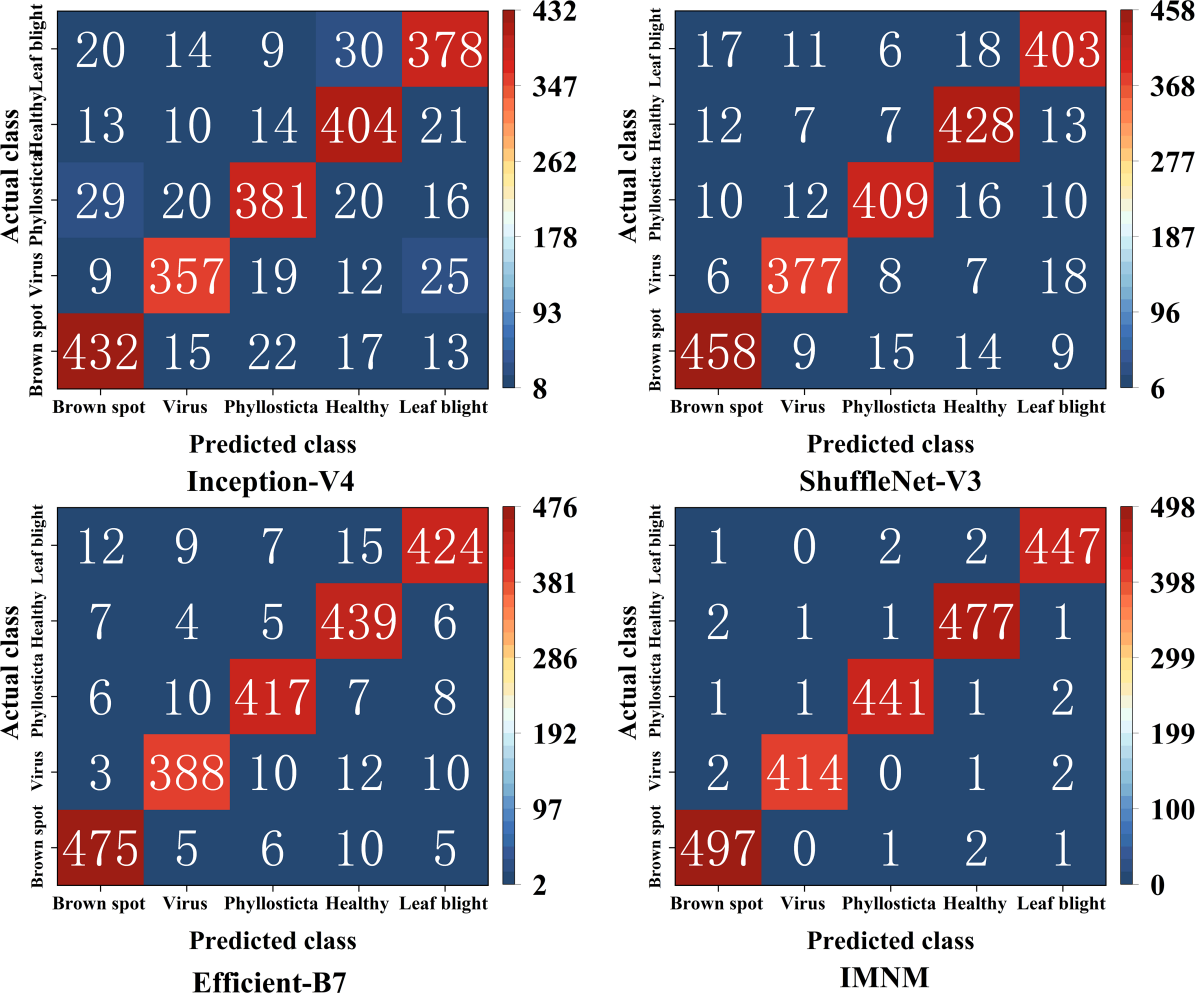
The classification results of the confusion matrices on the four models.

To present the predictive effect of the different models for each disease category, the study used a confusion matrix to create the classification results in **Figure 9**. Where the rows reflect a true category, the columns correspond to a unique prediction, and the secondary diagonal is the total number of correctly classified items.

#### 4.7.2 Comparison of the comprehensive performance of the above models

According to **Figure 9**, the study calculated that the overall identification accuracy of Inception-V4, ShuffleNet-V3, Efficient-B7, and IMNM was 84.95%, 90.16%, 93.16%, and 98.55%, respectively. As shown in **Table 3**, the IMNM exhibits a significant advantage in the overall performance of the model. Firstly, in terms of the balance between accuracy and efficiency, its accuracy is 5.39% higher than EfficientNet-B7, while the number of parameters is reduced by 61.9%, and the inference speed is increased by 4.8 times, realizing the organic unity of high accuracy and low calculation cost. Secondly, at the level of lightweight design, the FLOPs of the IMNM are only 57.6% of those of Inception-V4, and memory consumption is reduced by 40.4%, which significantly improves the deployment adaptability of the model on edge devices. These advantages reflect the advanced nature of the IMNM in model design and lay a solid foundation for its efficient application in actual agricultural disease identification scenarios.

**Table 3:** Comprehensive performance comparison.

Index	Inception-V4	ShuffleNet-V3	EfficientNet-B7	IMNM
Identification accuracy (%)	84.95	90.16	93.16	98.55
Parameter quantity (M)	42.30	5.80	66.40	25.30
FLOPs (G)	12.50	0.80	37.10	7.20
Inference time (ms)	45.20	18.70	92.50	19.30
GPU memory occupancy (GB)	5.20	1.50	8.70	3.10

#### 4.7.3 Analysis and discussion of the reasons for the performance differences of each model

##### 4.7.3.1 Efficiency bottleneck under the complex structure of Inception-V4

As shown in **Table 3**, Inception-V4 enhances feature extraction capability through multi-branch parallel convolution, but its complex architecture results in a significant increase in computational complexity (FLOPs 12.5G) and inference latency of up to 45.2ms. The static weight fusion strategy could not adapt to the texture characteristics of different diseases, and the final identification accuracy was only 84.95%. In addition, multi-branch output needs to store a large number of intermediate feature maps, and GPU memory occupies 5.2GB, which seriously limits its deployment feasibility in the mobile device field. Although its branching design supports multiscale analysis in theory, redundant computation and high memory requirements are the main bottlenecks in practical applications.

##### 4.7.3.2 ShuffleNet-V3 lightweight precision compromise

As shown in **Table 3**, ShuffleNet-V3 uses channel shuffling and deep separable convolution to achieve fast inference of 18.7ms with extremely low computational cost (FLOPs 0.8G), but its shallow network is difficult to extract high-level semantic features, resulting in insufficient discrimination ability for subtle lesions, with an accuracy rate of only 90.16%. Although the parameters are as low as 5.8M, the receptive field of a single convolution kernel limits the ability to capture multi-scale features and cannot balance the local details and global morphological differences of leaf diseases. Although this lightweight design is suitable for simple tasks on the mobile side, it is difficult to meet the high-precision identification requirements of complex diseases in agricultural scenarios.

##### 4.7.3.3 EfficientNet-B7 with high precision at a high cost

As shown in **Table 3**, EfficientNet-B7 optimizes depth, width and resolution uniformly through a compound scaling strategy, with a high accuracy rate (93.16%), but its deep network and large parameter volume (66.4M) cause a surge in computational load (FLOPs 37.1G), and inference speed as low as 92.5ms. More seriously, its sensitivity to small sample categories causes overfitting and limited generalization performance. In addition, the 8.7GB GPU memory footprint far exceeds the hardware ceiling of common edge devices, and the actual deployment cost is high, making it difficult to apply on a large scale in agricultural scenarios.

##### 4.7.3.4 Comprehensive breakthrough of the IMNM dynamic collaborative optimization

**Figure 10:**
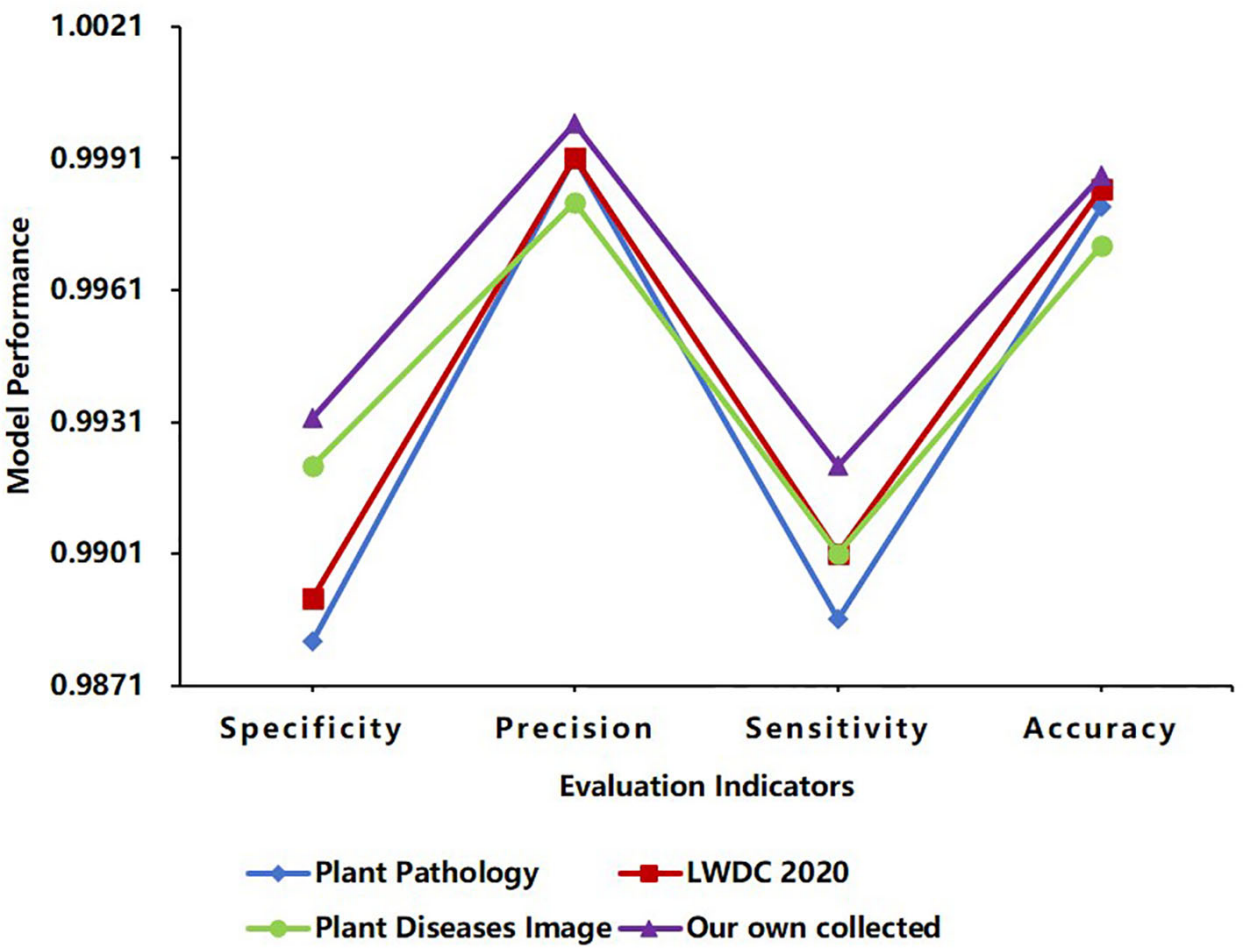
Comparisons of different databases using the IMNM.

As shown in **Table 3**, the IMNM achieves triple improvement in accuracy, efficiency, and generalization through collaborative design of the improved ResNet, DCN, and PPN. The DCN uses an attention mechanism to dynamically fuse multi-scale convolution kernels to accurately capture the characteristic differences between virus spots and leaf blight diffusion lesions. FLOPs are only 7.2 G, and the inference speed is 19.3 ms, which is close to the lightweight level of ShuffleNet-V3. The PPN optimizes training efficiency through prototype momentum updates and supports cross-crop generalization (as shown in **Figure 10**, average identification accuracy 99.81%). The IMNM (parameter size 25.3M) and memory optimization strategy (GPU memory consumption 3.1GB) make it compatible with full-platform deployment from server to edge equipment, providing cost-effective solutions for agricultural disease identification.

### 4.8 IMNM performance analysis based on violin graph

**Figure 11:**
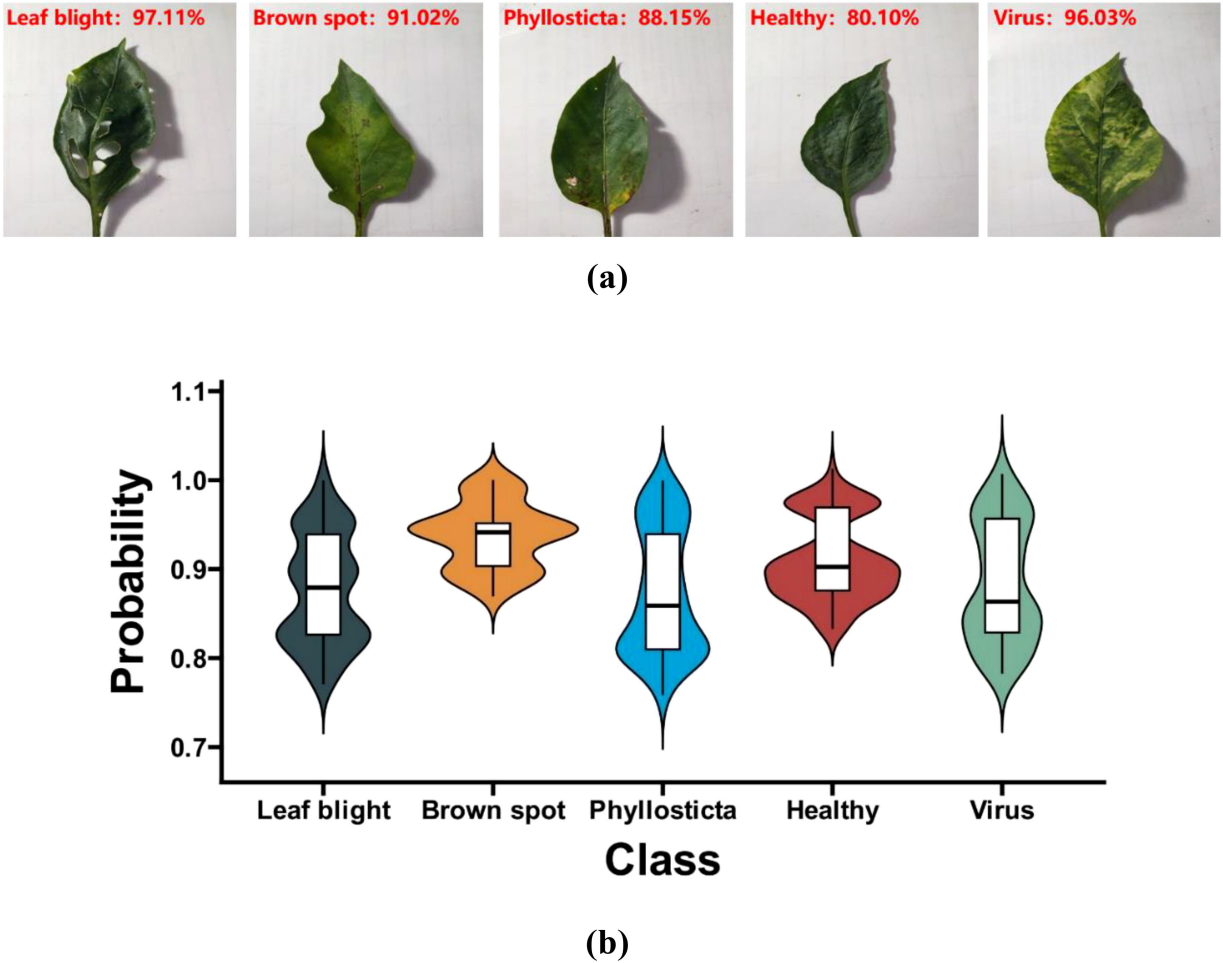
**(a)** The random test results; **(b)** The correct probability distribution violin chart.

This study used the IMNM to predict five different sets of randomly selected samples from the test set. The test results are presented in **Figure 11a**, where the left is the predicted class, and the right is the identification probability of the predicted class. In addition to observing the overall distribution of correctly identified probability values, this study used the violin chart to describe these identification probabilities in **Figure 11b**.

The results from **Figure 11a** indicate that the IMNM has an identification probability of over 70% for pepper leaf diseases. The results from **Figure 11b** indicate that the distribution of brown spot identification probability values performs best, as its median is the highest and its densest areas are the highest. In conclusion, the IMNM has high identification probabilities for the identification of pepper leaf diseases.

### 4.9 Verification of the generalization capabilities of the IMNM

To verify the generalization ability of the IMNM on other datasets, this study estimates its generalization ability from four aspects: Specificity, Precision, Sensitivity, and Accuracy.

#### 4.9.1 Details of other datasets

Three other datasets, Plant Pathology, LWDCD2020, and Plant Diseases Image, will be introduced below, detailing their leaf names, total number of samples, number of sample categories, number of samples in each category, data enhancement methods, image resolution, shooting equipment, shooting environment, and acquisition links. Their detailed information is shown in **Table 4**.

**Table 4:** Detailed statistics for other datasets.

Index	Plant Pathology	LWDCD2020^*^	Plant Diseases Image
Leaf name	Plant Pathology (Apple leaf)	LWDCD2020 (Wheat leaf)	Plant Diseases Image (Rice leaf)
Total sample size	3651	4990	5932
Number of sample categories	4	4	4
Size of samples per category	Apple scab:1200, Cedar apple rust:1399, Complex disease:187, Healthy:2008	Leaf rust:1620, Fusarium head blight: 1057, Crown and root rot: 1033, Healthy:1280	Bacterial blight:1584, Blast:1440, Brown spot:1600, Tungro:1308
Data enhancement means	Random lighting, upside-down flip, left-right flip, random rotation, and Gaussian blur, etc.	Rotation, scaling, width-to-height translation, clipping, horizontal flip, filling, etc.	Rotate left and right, flip vertically and horizontally, etc.
Image resolution	2048×1365	224x224	300x300
Photographing apparatus	Canon Rebel T5i DSLR and smartphone	Digital camera, but its model is unknown	Nikon DSLR-D5600 Camera
Shooting environment	Under various lighting, angles, surfaces, and noise conditions	Field collection	Different rice fields in Sambalpur and Bargarh districts of Odisha, India
Getting links	Please refer to the “Data availability statement” section of this paper	Please refer to the “Data availability statement” section of this paper	Please refer to the “Data availability statement” section of this paper

*For the LWDCD2020 datasets above, officially, there are 10 categories, whose total sample size is 12160, but only 4 categories above are publicly available, whose total sample size is 4990. So these 4 open categories above were used in this study.

#### 4.9.2 Generalization ability performance of the IMNM

As shown in **Figure 10**, the IMNM performed well on the other three datasets: Plant Pathology, LWDCD2020, and Plant Diseases Image. The results of the IMNM in the four evaluation indicators of Specificity, Precision, Sensitivity, and Accuracy were all greater than 98%, and the average identification accuracy reached 99.81%. These results show that the IMNM has good generalization ability.

#### 4.9.3 Analysis and discussion of the reasons for the excellent generalization performance of the IMNM

##### 4.9.3.1 The cross-domain feature coding mechanism of the improved ResNet

The improved ResNet constructs a hierarchical feature expression system through cross-layer residual connection and channel progressive expansion strategy, which effectively alleviates the gradient attenuation problem of deep networks and enables it to extract high-order semantic features with cross-crop invariance. The experimental data show that the Specificity of the IMNM is 0.9881, 0.9891, 0.9921 and 0.9932, respectively on Plant Pathology, LWDC 2020, Plant Diseases Image and Own datasets, and the range between them is only 0.0051, which indicates that the IMNM with the improved ResNet has strong generalization ability for extracting different plant disease features. In addition, the average identification accuracy of the IMNM on the four datasets reached 99.81%, indicating that the IMNM can achieve high-accuracy identification robustness under different crop disease scenarios, laying a solid feature foundation for cross-crop generalization.

##### 4.9.3.2 Multi-scale adaptive characteristics of the DCN

The DCN adaptively allocates the weights of multi-scale convolution kernels through an attention-driven multi-kernel fusion mechanism, and accurately matches different scale lesion features such as dense spots, diffuse lesions, and long-range morphology, which enables the IMNM with the DCN to dynamically adapt to feature extraction requirements in different scenes. As shown in **Table 3**, the computational load FLOPs of the IMNM with the DCN is 7.2G, which is only 19.4% of EfficientNet-B7. With such a low computational load, the IMNM has a Sensitivity mean of 0.9902, Sensitivity variance of only 1.55×10^-5^, Precision mean of 0.9993, Precision variance of only 4.08×10–^6^ across the dataset, as shown in **Figure 10**. This indicates that the IMNM has excellent Sensitivity and Precision performance across four datasets and is stable across datasets. In conclusion, the DCN provides the IMNM with a dynamic balance between computational efficiency and feature representation.

##### 4.9.3.3 Dynamic semantic alignment mechanism of the PPN

The PPN adopts a momentum prototype updating strategy to dynamically optimize semantic consistency across the crop feature space. By balancing historical prototype stability with freshness of current batch characteristics, the PPN maps significantly different disease patterns, such as rice blast and pepper brown spot, to a unified disease spread metric space. As shown in **Figure 10**, the IMNM with the fused PPN has an accuracy range of only 0.0016 across datasets, a specificity range of only 0.0051, and a sensitivity range of only 0.0035. This indicates that the Accuracy, Specificity, and Sensitivity performance of the IMNM on the four datasets does not fluctuate significantly. Therefore, this mechanism of the PPN systematically guarantees the generalization reliability of the IMNM in complex agricultural scenarios and provides a theoretical guarantee for real-time identification of multiple crop diseases.

### 4.10 Performance comparison based on recent published literature methods and the IMNM

To further confirm the performance of IMNM, this study collected recent published literature methods for comparative analysis. **Table 5** provides details of the comparative analysis.

**Table 5:** Comparison of the IMNM with recent published literature methods.

Ref., year	Methods	Dataset size	The number of classes	Identification accuracy
Gopinath et al., 2022	CRNN	1496	2	94.14%
Naik et al., 2022	SE-CNN	4590	5	99.12%
Dai et al., 2023	Lightweight CNN	9183	6	97.87%
Begum and Syed, 2024	GSAtt-CMNetV3	1855	2	97.87%
Proposed, 2024	IMNM	11447	5	98.55%

According to **Table 5**, the IMNM has an accuracy rate that is 0.57% lower than the state-of-the-art method by Naik et al. (2022). Furthermore, the identification accuracy of the IMNM increased by 4.41% when compared to the method by Gopinath et al. (2022). In conclusion, the recent literature indicates that the IMNM has high effectiveness in identifying pepper leaf diseases.

## 5 Conclusions and outlooks

### 5.1 Conclusion

In this study, five different types of pepper leaves were utilized as samples to train, validate, and test the accuracy of the IMNM classification. To extract the fundamental features of pepper leaf disease, first, the pepper leaf disease image is sent to the improved ResNet; second, the features of important regions are extracted by the DCN, and the appropriate convolution kernel is adaptively selected; finally, the feature distribution and training efficiency are optimized through the PPN. Experimental results indicate that the IMNM model achieves an identification accuracy of 98.55%, with specificity, precision, and sensitivity surpassing those of Inception-V4, ShuffleNet-V3, and Efficient-B7. Furthermore, the model demonstrated strong generalization capabilities, achieving an average identification accuracy of 99.81% in identifying leaf diseases in apple, wheat, and rice, thereby confirming the effectiveness of multi-scale feature fusion and dynamic adaptive design. In short, the IMNM has good performance in balance of the lightweight and the high identification accuracy, simultaneous improvement of the dynamic feature extraction capability and the computational efficiency, trade-offs between the GPU memory occupancy and the edge deployment adaptability, simultaneous improvement of the cross-crop generalization ability and the high identification accuracy, and collaborative optimization of identification accuracy and multi-network fusion quality. The main innovation points of the IMNM are summarized based on **Table 3**, **Table 5**, and relevant contents mentioned above, as shown in **Table 6**.

**Table 6:** Summary of main innovation points of the IMNM.

Main innovation points of the IMNM	Performance indicators of the comparative models/literature models	Performance indicators of the IMNM	The comparison of performance indicators
Balance of the lightweight and the high identification accuracy	The parameter quantity of EfficientNet-B7 is 66.4M, its identification accuracy is 93.16%, and its inference time is 92.50ms.	Its parameter quantity is 25.30 M. Its identification accuracy is 98.55%. Its inference time is 19.30ms.	Compared with EfficientNet-B7, the IMNM reduces the parameter quantity by 61.90%, but improves the identification accuracy by 5.39%. The inference speed is 4.79 times faster than EfficientNet-B7.
Simultaneous improvement of the dynamic feature extraction capability and the computational efficiency	The FLOPs of the Inception-V4 are 12.50G, and its inference time is 45.20ms. The FLOPs of the EfficientNet-B7 are 37.10G, and its inference time is 92.50ms.	Its FLOPs are 7.20G. Its inference time is 19.30ms.	With the help of the IMNM with the integrated DCN design, the IMNM can reduce FLOPs by 42.40% and increase inference speed by 2.34 times compared with Inception-V4, and reduce FLOPs by 80.60% and increase the inference speed by 4.79 times compared with Efficient Net-B7.
Trade-offs between the GPU memory occupancy and the edge deployment adaptability	The GPU memory occupancy of the EfficientNet-B7 is 8.70GB, and its identification accuracy is 93.16%.	Its GPU memory occupancy is 3.10 GB. Its identification accuracy is 98.55%.	Compared with EfficientNet-B7, the IMNM has an identification accuracy increase of 5.39%, but the GPU memory occupancy is reduced by 64.37%. The IMNM can better adapt to edge devices such as Jetson Nano (4GB memory).
Simultaneous improvement of the cross-crop generalization ability and the high identification accuracy	The SE-CNN of Naik et al. (2022) supports 5 classes with an identification accuracy of 99.12%. The GSAtt-CMNetV3 of Begum and Syed (2024) supports Class 2 with an identification accuracy of 97.87%.	Supporting 5 kinds of pepper leaves, its identification accuracy is 98.55%. It can identify other crops, such as apple, rice, and wheat leaves, and its average identification accuracy is 99.81%.	The IMNM supports more categories (5 categories). The generalization ability of the IMNM across crops was significantly enhanced by introducing the PPN momentum update prototype. The identification accuracy of the IMNM is only 0.58% lower than Naik’s SE-CNN, but 0.70% higher than Begum’s GSAtt-CMNetV3.
Collaborative optimization of identification accuracy and multi-network fusion quality	The identification accuracy of the ShuffleNet-V3 is 90.16%, and its inference time is 18.70ms	Its identification accuracy is 98.55%. Its inference time is 19.30ms It fuses the improved ResNet, DCN, and PPN.	When the inference time difference between the IMNM and the ShuffleNet-V3 is small, the identification accuracy of the IMNM is 8.39% higher than that of the ShuffleNet-V3. In addition, the ablation experiments in Section 4.6 show that the identification accuracy of the IMNM with the multi-network fusion is 24.92% higher than that of a single PPN.

### 5.2 Outlooks

Building on current research, future work targets three key areas: model reliability, interpretability, and engineering implementation challenges. The details are as follows.

#### 5.2.1 Study on reliability and module cooperation of multi-source heterogeneous data enhanced model

Expanding the sample size, improving the balance of data distribution, enhancing the database of multi-source heterogeneous diseases, and improving the quality of data labeling to ensure the reliability of the model. This will involve integrating data from 3D imaging (Zhou et al., 2024), multi-spectral analysis (He et al., 2025), multi-spectral imaging (He et al., 2025), real-time wireless sensing (Wang et al., 2023), microorganisms (Han et al., 2024), modern sequencing and genomics (Wang M. et al., 2024), and so on. The goal is to increase sensitivity to early, novel, and rare diseases while verifying the module synergy mechanism through statistical tests, standard deviation analysis, and ablation experiments.

#### 5.2.2 Study on model interpretability enhancement and decision tool development for multimodal data fusion

Advancing research on multi-modal interpretability by combining 3D point cloud data (Zhou et al., 2024), thermal diagram visualization, and causal reasoning technology. This will lead to the development of user-friendly interactive decision-making tools for farmers, thereby improving model logic transparency and biological relevance.

#### 5.2.3 Realistic challenges, technical limitations, ethical considerations, moving photoing adaptability, and future application deployment plans in model implementation

##### 5.2.3.1 Realistic challenges include equipment adaptation and ease of operation

Uneven coverage of rural networks affects model invocation, while the limited computing power of low-cost equipment leads to low efficiency of traditional models. Additionally, some farmers lack the necessary skills for operation and require a lower threshold. Future research plans: lightweight model to achieve rapid reasoning of low-computing devices; development of offline mode to support network disconnection identification; minimal interface integration of dialect guidance and training to improve proficiency.

##### 5.2.3.2 The technical limitation is the problem of data coverage and equipment adaptation

the current model can only identify 5 pepper leaf types, the expansion ability is limited, and the network structure needs to adapt to new categories; the disease coverage of the dataset is insufficient, and the field environmental variables affect the identification stability; the traditional model has high power consumption and large cross-equipment adaptation cost. Future research plans: optimization by factor graph (Xiao et al., 2024), multimodal data fusion (Cai et al., 2025) and dynamic threshold adjustment to implement multi-scenario data enhancement, expand data and improve robustness; dynamic calculation to optimize matching equipment calculation; establishment of compatibility test system customization optimization to reduce deployment difficulty.

##### 5.2.3.3 Ethical considerations are data security and compliance risk issues

data security and compliance risk. Identification results contain sensitive production data with a risk of leakage/abuse; training data management needs to avoid disputes. Future research plans: end-to-end encryption, local processing does not transmit the original image, only returns the identification suggestion; establishes the data ledger to clarify the purpose; the technical certificate guarantees source traceability and processing transparency.

##### 5.2.3.4 Moving photoing adaptability

The 98.55% identification accuracy of this study was obtained when the camera was stationary. To test the inference effect of the IMNM in the case of moving the mobile phone camera to photo, this study conducted the test in the laboratory. The following are the test processes:

① Use the electric slide to control the Honor Play 4 mobile phone camera to take photos of pepper leaves at moving speeds of 0.1m/s, 0.3m/s, and 0.5m/s, respectively.
② Inputting the photos into the IMNM program for inference, the identification accuracy of the IMNM at three speeds is 98.53%, 98.42%, and 98.23%, respectively, which is only 0.02%, 0.13%, and 0.32% lower than that of 98.55% of static reference, respectively.
③ Concluding from the test, the IMNM is robust to motion disturbances when moving photoing inference is performed at the above moving speeds, and more tests will be carried out in the future to illustrate the issue of the moving photoing adaptability of the IMNM.

##### 5.2.3.5 Future application deployment plans

###### 5.2.3.5.1 Rapid deployment stage

The government deploys the IMNM application-based Cloud Server in areas with perfect network infrastructure and develops an IMNM-based mobile phone APP. Farmers obtain identification results through personal smartphones in the field with the help of this APP.

###### 5.2.3.5.2 Transitional implementation stage

In areas with poor network stability or low smartphone penetration, the government configures agricultural stores with mobile devices pre-installed with the offline-optimized IMNM applications, with which farmers can obtain identification results.

###### 5.2.3.5.3 Local deployment stage

The government introduced edge computing devices embedded with the optimized IMNM applications in villages without network services but with a stable power supply, allowing farmers to quickly obtain identification results.

###### 5.2.3.5.4 High-performance service stage

The government provides high-performance mobile devices integrated with the optimized IMNM applications to large-scale growers with high identification accuracy requirements, which farmers can use to quickly obtain identification results in real time in the field.

###### 5.2.3.5.5 All regional intelligent services stage

The government will build cloud and edge collaborative identification service networks based on the IMNM in mixed agricultural areas with significant technical differences, and provide dedicated, ubiquitous, ultra-high-performance handheld identification terminals for farmers who do not have mobile phones. Regardless of whether farmers have mobile phones or their mobile phones and network status, the system or the above terminals will provide farmers with ultra-high-quality identification services in all regions.

## Data Availability

The data presented in the study are deposited in the Figshare repository, accession number 10.6084/m9.figshare.29400611.

## References

[B1] Ashurov A. Y. Al-Gaashani M. S. Samee N. A. Alkanhel R. Atteia G. Abdallah H. A.. (2025). Enhancing plant disease detection through deep learning: A Depthwise CNN with squeeze and excitation integration and residual skip connections. Front. Plant Sci. 15, 1505857. doi: 10.3389/fpls.2024.1505857, PMID: 39925367 PMC11803862

[B2] Begum S. Syed H. (2024). GSAtt-CMNetV3: pepper leaf disease classification using osprey optimization. IEEE Access 12, 32493–32506. doi: 10.1109/ACCESS.2024.3358833

[B3] Bhar A. Chakraborty A. Roy A. (2022). Plant responses to biotic stress: old memories matter. Plant-Basel 11, 84. doi: 10.3390/plants11010084, PMID: 35009087 PMC8747260

[B4] Bhuma C. Kongara R. (2022). Virus texture classification of TEM images using fusion of chebyshev moments and resnet50 features. Braz. Arch. Biol. Technol. 65, e22210636. doi: 10.1590/1678-4324-2022210636

[B5] Cai G. Zheng X. Guo J. Gao W. (2025). Real-time identification of borehole rescue environment situation in underground disaster areas based on multi-source heterogeneous data fusion. Saf. Sci. 181, 106690. d1ay01726h. doi: 10.1016/j.ssci.2024.106690

[B6] Chaudhari V. V. Patil M. P. (2023). Detection and classification of banana leaf disease using novel segmentation and ensemble machine learning approach. Appl. Comput. Syst. 28, 92–99. doi: 10.2478/acss-2023-0009

[B7] Chen Y. Dai X. Liu M. Chen D. Yuan L. Liu Z. (2020). “Dynamic convolution: Attention over convolution kernels,” in Proceedings of the IEEE/CVF Conference on CVPR 2020. Seattle, WA, USA: IEEE Computer Society / CVF.

[B8] Dai M. Sun W. Wang L. Dorjoy M. Zhang S. Miao H.. (2023). Pepper leaf disease recognition based on enhanced lightweight convolutional neural networks. Front. Plant Sci. 14. doi: 10.3389/fpls.2023.1230886, PMID: 37621882 PMC10445539

[B9] Fakhrurroja H. Hidayatullah A. R. Pramesti D. Ismail N. (2024). “Classification of diseases in chili plants using the SVM method: development and implementation,” in 2024 12th International Conference on ICoICT. Yogyakarta, Indonesia: IEEE.

[B10] Gautam V. Trivedi N. K. Singh A. Mohamed H. G. (2022). A transfer learning-based artificial intelligence model for leaf disease assessment. Sustainability 14, (20). doi: 10.3390/su142013610

[B11] Gopinath S. Sakthivel K. Lalitha S. (2022). A plant disease image using convolutional recurrent neural network procedure intended for big data plant classification. J. Intelligent Fuzzy Syst. 43, 4173–4186. doi: 10.3233/JIFS-220747

[B12] Han Y. Huang G. Song S. (2022). Dynamic neural networks: A survey. IEEE Trans. Pattern Anal. Mach. Intell. 44, 7436–7456. doi: 10.1109/TPAMI.2021.3117837, PMID: 34613907

[B13] Han Y. Zhang Y. Yang Z. Zhang Q. He X. Song Y.. (2024). Improving aerobic digestion of food waste by adding a personalized microbial inoculum. Curr. Microbiol. 81, 277. doi: 10.1007/s00284-024-03796-5, PMID: 39028528

[B14] He Q. Zhan J. Liu X. Dong C. Tian D. Fu Q. (2025). Multispectral polarimetric bidirectional reflectance research of plant canopy. Optics Lasers Eng. 184, 108688. doi: 10.1016/j.optlaseng.2024.108688

[B15] He K. Zhang X. Ren S. Sun J. (2016). “Deep residual learning for image recognition,” in Proceedings of the IEEE Conference on CVPR 2016. Las Vegas, NV, USA: IEEE Computer Society.

[B16] Islam S. Samsuzzaman Reza M. N. Lee K.-H. Ahmed S. Cho Y. J.. (2024). Image processing and support vector machine (SVM) for classifying environmental stress symptoms of pepper seedlings grown in a plant factory. Agronomy 14, 2043. doi: 10.3390/agronomy14092043

[B17] Kim S. G. Lee S.-D. Lee W.-M. Jeong H.-B. Yu N. Lee O.-J.. (2025). Effective tomato spotted wilt virus resistance assessment using non-destructive imaging and machine learning. Horticulturae 11, 132. doi: 10.3390/horticulturae11020132

[B18] Naik B. Malmathanraj R. Palanisamy P. (2022). Detection and classification of chilli leaf disease using a squeeze-and-excitation-based CNN model. Ecol. Inf. 69, 101663. doi: 10.1016/j.ecoinf.2022.101663

[B19] Omae N. Tsuda K. (2020). Plant-microbiota interactions in abiotic stress environments. Mol. Plant-Microbe Interact. 35, 511–526. doi: 10.1094/MPMI-11-21-0281-FI, PMID: 35322689

[B20] Palve V. (2023). Advanced deep learning techniques for plant disease diagnosis and treatment. Int. J. Res. Appl. Sci. Eng. Technol. 11, 3372–3379 doi: 10.22214/ijraset.2023.56753

[B21] Rusliyawati R. Karnadi K. Tanniewa A. M. Widyawati A. C. Jusman Y. Borman R. I. (2024). Detection of pepper leaf diseases through image analysis using radial basis function neural networks. Bio Web Conferences. 97, 00122. doi: 10.1051/bioconf/202414401005

[B22] Savary S. Willocquet L. Pethybridge S. (2019). The global burden of pathogens and pests on major food crops. Nat. Ecol. Evol. 3, 430–439. doi: 10.1038/s41559-018-0793-y, PMID: 30718852

[B23] Shaheen N. Khan U. Azhar M. (2021). Genetics and genomics of fusarium wilt of chilies: A review. Agronomy-Basel 11, 2162. doi: 10.3390/agronomy11112162

[B24] Shaikh T. Rasool T. Lone F. (2022). Towards leveraging the role of machine learning and artificial intelligence in precision agriculture and smart farming. Comput. Electron. Agric. 198, 07119. doi: 10.1016/j.compag.2022.107119

[B25] Sultan T. Chowdhury M. S. Jahan N. Mridha M. Alfarhood S. Safran M.. (2025). LeafDNet: transforming leaf disease diagnosis through deep transfer learning. Plant Direct 9, e70047. doi: 10.1002/pld3.70047, PMID: 39943923 PMC11815709

[B26] Valderrama Solis M. A. Valenzuela Nina J. Echaiz Espinoza G. A. Yanyachi Aco Cardenas D. D. Villanueva J. M. M. Salazar A. O.. (2025). Innovative machine learning and image processing methodology for enhanced detection of aleurothrixus floccosus. Electronics 14, 358. doi: 10.3390/electronics14020358

[B27] Wan L. Zhu W. Dai Y. Zhou G. Chen G. Jiang Y.. (2024). Identification of pepper leaf diseases based on TPSAO-AMWNet. Plants 13, 1581. doi: 10.3390/plants13111581, PMID: 38891389 PMC11174783

[B28] Wang M. Lin H. Lin H. Du P. Zhang S. (2024). From species to varieties: how modern sequencing technologies are shaping Medicinal Plant Identification. Genes 16, 16. doi: 10.3390/genes16010016, PMID: 39858563 PMC11765323

[B29] Wang C. Min S. Chen X. Sun X. Li H. (2021). Dual progressive prototype network for generalized zero-shot learning. Adv. Neural Inf. Process. Syst. 34, 2936–2948. doi: 10.48550/arXiv.2111.02073

[B30] Wang T. Shen F. Deng H. Cai F. Shen S. (2022). Smartphone imaging spectrometer for egg/meat freshness monitoring. Analytical Methods 14, 508–517. doi: 10.1039/D1AY01726H, PMID: 35050274

[B31] Wang Y. Yin Y. Li Y. Qu T. Guo Z. Peng M.. (2024). Classification of plant leaf disease recognition based on self-supervised learning. Agronomy 14, 500. doi: 10.3390/agronomy14030500

[B32] Wang M. Zhang R. Wu Z. Xiao X. (2023). Flexible wireless in *situ* optical sensing system for banana ripening monitoring. J. Food Process Eng. 46, e14474. doi: 10.1111/jfpe.v46.12

[B33] Xiao G. Yang C. Wei H. Xiao Z. Zhou P. Li P.. (2024). PPP ambiguity resolution based on factor graph optimization. GPS Solutions 28, 178. doi: 10.1007/s10291-024-01715-6

[B34] Xu L. Su J. Li B. Fan Y. Zhao J. (2024). Spinach leaf disease identification based on deep learning techniques. Plant Biotechnol. Rep. 18, 953–965. doi: 10.1007/s11816-024-00944-y

[B35] Zhou Y. Zhou H. Chen Y. (2024). An automated phenotyping method for Chinese Cymbidium seedlings based on 3D point cloud. Plant Methods 20, 151. doi: 10.1186/s13007-024-01277-1, PMID: 39343899 PMC11441005

